# Effects of aspirin, vitamin D3, and progesterone on pregnancy outcomes in an autoimmune recurrent spontaneous abortion model

**DOI:** 10.1590/1414-431X2020e9570

**Published:** 2021-06-14

**Authors:** Yihong Chen, Qiumei Wu, Juanbing Wei, Jifen Hu, Shan Zheng

**Affiliations:** 1 Department of Obstetrics and Gynecology, The First Affiliated Hospital of Fujian Medical University, Fuzhou, China; 2 Department of Ultrasound, Fujian Provincial Maternal and Children's Hospital, Affiliated Hospital of Fujian Medical University, Fuzhou, China

**Keywords:** Autoimmune RSA, Cytokines, Aspirin, Vitamin D3, Progesterone

## Abstract

High proportions of placental lymphocytes expressing DX5+/CD25+/FOXP3+/CD45+/CD4+ are beneficial to maintain immune tolerance and improve pregnancy outcomes. This study aimed to compare and evaluate the therapeutic effects of aspirin, vitamin D3 (VitD3), and progesterone on the autoimmune recurrent spontaneous abortion (RSA) model. The autoimmune RSA mouse model was constructed, and the embryo loss rate was calculated for each group. Then, primary mouse placental lymphocytes were isolated, and the expression of DX5+/CD25+/FOXP3+/CD45+/CD4+ was detected through flow cytometry. The serum levels of anti-cardiolipin antibody (ACA), β2-GP1, CXCL6, IFN-γ, and IL-6 were measured by ELISA to evaluate the proportion of Th1 and Th2 cells. Autoimmune RSA significantly increased the embryo loss rate, which was improved by aspirin, VitD3, and progesterone treatment, and progesterone treatment had the best effect among the three treatments. The positive expression of DX5+/CD25+/FOXP3+/CD45+/CD4+ in the VitD3 and progesterone groups was significantly higher than that in the autoimmune RSA group, and the expression was highest in the progesterone treatment group. In the plasma of autoimmune RSA mice, the ACA, β2-GP1, CXCL6, and IFN-γ levels were significantly higher and the IL-6 level was lower than the levels in control mice. All these changes could be reversed by aspirin and progesterone treatment. In conclusion, aspirin, VitD3 and progesterone treatment improved pregnancy outcomes in autoimmune RSA mice by regulating the Th1/Th2 balance and cytokines, and progesterone had the best effect of the three treatments.

## Introduction

Spontaneous abortion is a common complication in the gestational period and is generally referred to as a pregnancy failure since it occurs before 28 weeks of gestation and fetal weight less than 1000 g (1,2). Recurrent spontaneous abortion (RSA) is the occurrence of spontaneous abortion at least three times in succession (3). Apart from chromosomal, anatomic, and endocrine abnormalities, approximately 50-60% of RSAs are related to immune factors in terms of its pathogenesis (4,5). Patients with autoimmune spontaneous abortion have one or multiple autoantibodies, including anti-cardiolipin antibody (ACA), anti-zona pellucida antibody (AzpAb), anti-ovarian antibody (AoAb), and anti-endometrium antibody (EmAb) (6,7). Among the autoantibodies, ACA is the most highly correlated with RSA, likely inducing placental thrombosis and obstruction, leading to abortion (8). Research indicates that ACAs can bind with phospholipids on the vascular endothelial cell surface to form immune complex deposits and induce a series of inflammatory changes that ultimately lead to thrombosis (9,10).

Aspirin, the primary antiplatelet drug, can irreversibly suppress epoxidase-1 activity in order to exert its anti-platelet aggregation effect. Aspirin can be hydrolyzed into salicylic acid and acetic acid *in vivo* and can effectively inhibit abortion of an embryo without causing any adverse reactions in pregnant women (11,12). At present, the main treatment for autoimmune RSA is aspirin combined with heparin. Vitamin D3 (VitD3) is a type of steroid derivative that can be involved in immune regulation through cellular signal transduction pathways; in addition, VitD3 may also be related to T-lymphocyte differentiation and induction of immune tolerance, but its role in spontaneous abortion remains unclear (13,14). Progesterone, a natural progestogen secreted by the ovary, is the first-line drug for clinical luteal support and confers obvious protection for the endometrium. Its common clinical indications include premature delivery, dysfunctional uterine bleeding, assisted reproduction after embryo transplantation, and luteal phase defects. Progesterone has no androgen activity, few side effects, and favorable clinical safety (15,16).

In this study, anti-β2-GP1 antibodies and ACAs were induced by injecting mice with β2-GP1 to construct the autoimmune RSA mouse model (17). Based on the current understanding of that disease model, aspirin, VitD3, and progesterone were used to intervene in the pregnancy outcome of autoimmune RSA. Additionally, the potential effects, advantages, and disadvantages of the three drugs were also examined to provide a reference for further research.

## Material and Methods

### Animals

BALB/c mice (female, 20±2.05 g, 54-58 days old; male, 26±3.42 g, 47-51 days old) were purchased from Changsha SLAC Laboratory Animal Company [China, http://www.hnsja.com/; Certificate No. SCXK (Xiang) 2016-0002]. All animals were housed in cages with free access to food and water, and they were acclimated for 1 week of adaptive feeding under conditions of 24°C on a normal 12-h light/dark cycle (lights on at 7:00 AM) prior to experimental surgery to minimize animal suffering.

The animal protocol was approved by the Animal Care and Use Committee of The First Affiliated Hospital of Fujian Medical University and was consistent with the National Institutes of Health Guide for the Care and Use of Laboratory Animals.

### Reagents

β2-GP1 was purchased from Novoprotein (Cat. No. DC432, USA). Incomplete Freund's adjuvant (IFA) (Cat. No. 642862) and complete Freund's adjuvant (CFA) (Cat. No. 642852) were purchased from Solarbio (China). Progesterone was purchased from Solarbio (Cat. No. SP9440). Aspirin enteric-coated tablets were purchased from Bayer HealthCare (Cat. No. J20171021, USA). Vitamin D3 was purchased from Solarbio (Cat. No. V8070). A mouse spleen lymphocyte separation reagent kit was purchased from TBD Sciences (LTS1092PK, China). A mouse β2-GPI ELISA kit (Cat. No. MM-0951 M1), mouse interferon (IFN)-γ ELISA kit (Cat. No. MM-0182 M1), mouse ACA ELISA kit (Cat. No. MM-0583 M1), mouse interleukin (IL)-6 ELISA kit (Cat. No. MM-0163 M1), and mouse CXCL6 ELISA kit (Cat. No. MM-0950 M1) were all purchased from Meimian Biotechnology (China). CD4-FITC (Cat. No. 100405), CD25-PE/CY7 (Cat. No. 102015), CD49b-PE (Cat. No. 108907), FOXP3-Alexa Fluor 647 (Cat. No. 320013), CD45-Percp/CY5.5 (Cat. No. 103131), fixation buffer (Cat. No. 420801), and intracellular staining permeabilization wash buffer (Cat. No. 421002) were all purchased from BioLegend (USA).

### Autoimmune RSA mouse model

The autoimmune RSA mouse model was constructed according to the method described by Xiao et al. (7). Female mice were given intrauterine injections of 50 μL PBS containing 10 µg human β2-GP1 and 10 μg CFA for 1 week. Then, CFA was replaced with IFA, and the same dose was administered. After 10 days of injections, female mice were housed with male mice at a ratio of 2:1. From the date that cohabitation began, mice were observed at two fixed time periods in the morning and in the afternoon. If a vaginal plug was observed, the female was raised in an individual cage and was used for the autoimmune spontaneous abortion mouse model; the non-pregnant female mice were excluded. The day of vaginal plug detection was considered day 0 of pregnancy. A total of 35 pregnant female mice were randomly divided into 5 groups (n=7 for each group): the normal pregnant group (Control), the model group (Model, autoimmune RSA mice given distilled water by oral gavage at 0.1 mL/10 g once a day for 14 days continuously after the vaginal plug was observed), the aspirin group (Aspirin, autoimmune RSA mice given aspirin by oral gavage at 0.0195 mg/g once a day for 14 days continuously from the first day of gestation), the vitamin D3 group (VitD3, autoimmune RSA mice given VitD3 by intraperitoneal injection at 4 µg/mouse once a day for 14 days continuously from the first day of gestation), and the progesterone group (Progesterone, autoimmune RSA mice given progesterone by intraperitoneal injection at 4 mg/kg once a day for 14 days continuously from the first day of gestation).

On day 15 of gestation, blood samples (1 mL) were collected from the mouse abdominal aorta under anesthesia. Then, mice were sacrificed by cervical dislocation, and uterine horns were isolated from pregnant mice. The placental weight, fetal weight, and total number of embryos lost were recorded. The embryo loss rate was calculated according to the following formula: embryo loss rate (%) = (number of lost fetuses / number of fetuses) × 100.

### Placental lymphocyte isolation

The pregnant mice were sacrificed, disinfected with ethanol for 5 min, and dissected on the benchtop to collect the placentas. Afterwards, the placentas were cut into pieces and washed with Hanks' buffer 2-3 times. Then, the upper layer of the cell suspension was discarded, and 0.1% collagenase I was added into stop buffer to digest the tissue for 30 min, followed by agitation. The above steps were repeated several times until the placental tissues were completely digested. The cell suspension was filtered using a 70 µm sieve, 0.125% trypsin was used to digest the cells for 1-2 min, the suspension underwent centrifugation at 1480 *g* for 5 min at 4°C, and the monocytes were isolated using mouse visceral lymphocyte separating medium.

### Detection of DX5+/CD25+/FOXP3+/CD45+/CD4+ expression in placental lymphocytes by flow cytometry

A total of 100 µL cell suspension was collected in an Eppendorf tube, then cells were incubated with 5 µL each CD4-FITC, CD25-PE/CY75, CD49b-PE, and CD45-Percp/CY5.5 in the Eppendorf tube for 10 min in the dark. Afterwards, cells were washed in 1 mL PBS and fixed in 500 µL fixing agent for 30 min. Cells were then collected and washed in 1 mL PBS and incubated in 1 mL permeabilization reagent for 30 min in the dark. Subsequently, the cells were washed again and resuspended in 100 µL PBS with 5 µL FOXP3-Alexa Fluor 647 and incubated for 10 min in the dark. Cells were washed again and incubated in 500 µL PBS for analysis by flow cytometry (NovoCyte 2060R, Acea Biosciences, China). A total of 10,000 cells were screened and analyzed automatically by the flow cytometer, and the operator was blind to the group assignment of the cells.

### Enzyme-linked immunosorbent assay (ELISA)

The plasma levels of ACA, β2-GP1, CXCL6, IFN-γ, and IL-6 were determined using ELISA. In brief, blood samples (1 mL) were collected through the mouse's heart under deep anesthesia and then underwent 15 min of centrifugation at 800 *g* at 4°C. Then, the plasma IL-1β concentration was determined using commercially available ELISA kits in accordance with the manufacturer's instructions. Absorbance was measured at 450 nm using a microplate reader (RT-6100, Rayto Life and Analytical Sciences, China). The concentrations of each cytokine were calculated according to the standards and reported as picograms per milligram (pg/mg).

### Statistical analysis

Three repeated experiments were conducted for each set of measurements, and the resulting data are reported as means±SD. The statistical package SPSS 18.0 (IBM, USA) was used for all statistical analyses. The data were analyzed using one-way analysis of variance (ANOVA) followed by Dunnett's test. P<0.05 was considered statistically significant.

## Results

### Detection of embryo loss rate

**Figure 1:**
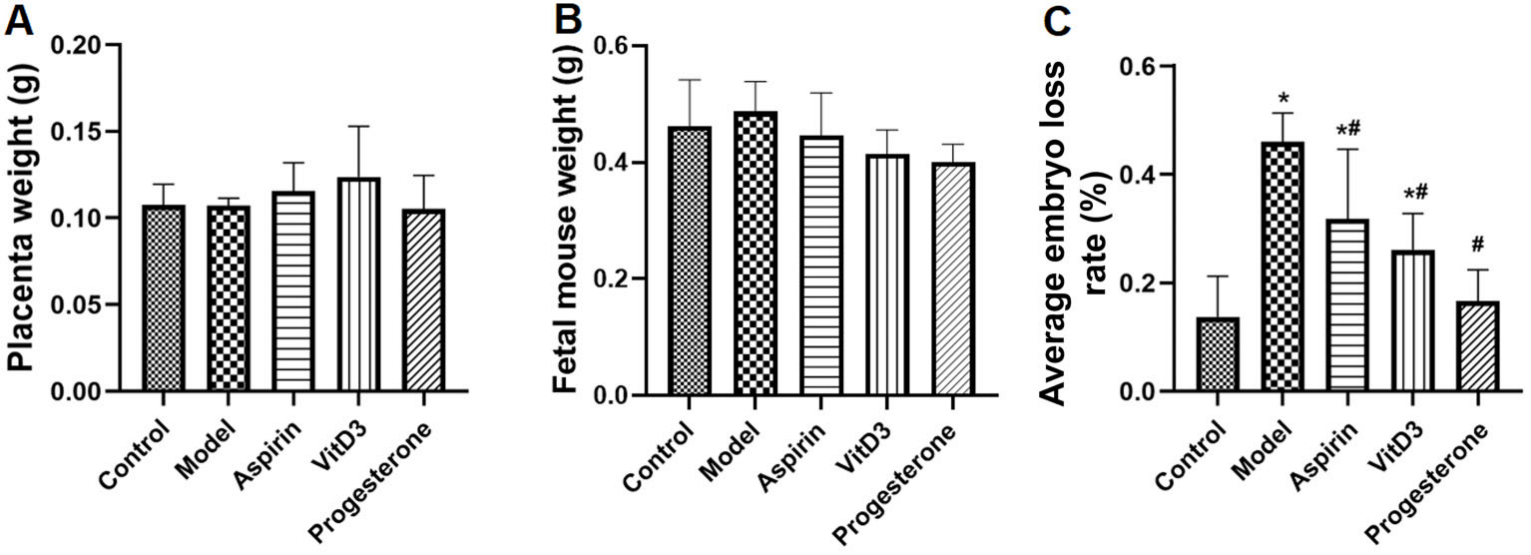
Effects of aspirin, vitamin D3 (VitD3), and progesterone on the placental weight (**A**), fetal mouse weight (**B**), and embryo loss rate (**C**) in autoimmune recurrent spontaneous abortion mice. Data are reported as means±SD (n=5). *P<0.05 *vs* the control group; ^#^P<0.05 *vs* the model group (ANOVA).

Pregnancy outcomes are shown in Figure 1. There were no significant differences in the placental weight and fetal mouse weight among the groups. Autoimmune RSA significantly increased the embryo loss rate, which was improved by treatment with aspirin, VitD3, and progesterone; progesterone treatment had the best effect.

### Detection of DX5+/CD25+/FOXP3+/CD45+/CD4+ expression in placental lymphocytes

**Figure 2:**
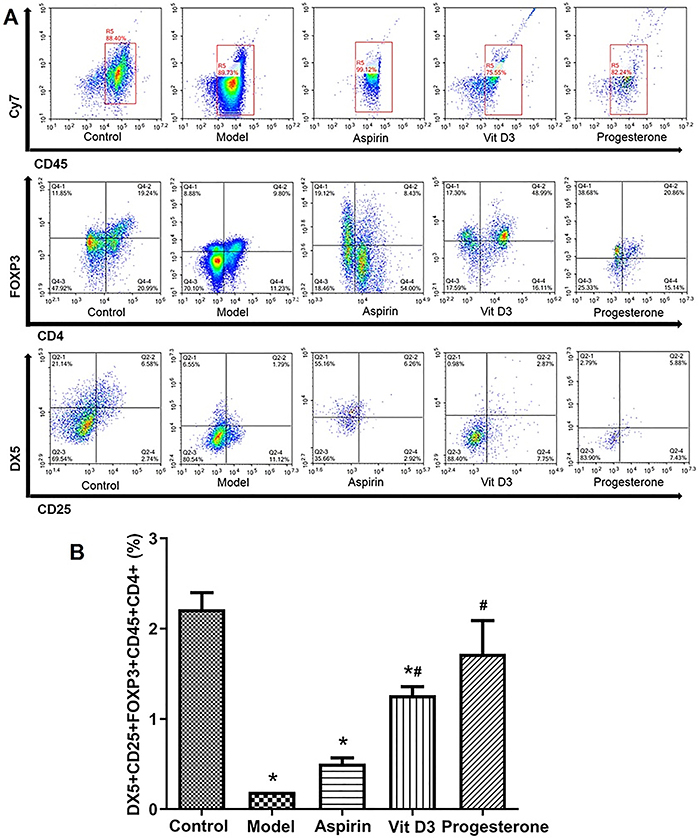
The expression of DX5+/CD25+/FOXP3+/CD45+/CD4+ in placental lymphocytes in a mouse model of recurrent spontaneous abortion treated with aspirin, vitamin D3, and progesterone was detected by flow cytometry. **A**, Placental lymphocytes were collected and stained with relevant markers for flow cytometry analysis. **B**, Quantitative analysis of the percentage of DX5+/CD25+/FOXP3+/CD45+/CD4+ cells in each group. Data are reported as means±SD (n=3). *P<0.05 *vs* the control group; ^#^P<0.05 *vs* the model group (ANOVA).

The high percentage of DX5+/CD25+/FOXP3+/CD45+/CD4+-positive placental lymphocytes is beneficial to maintain immune tolerance and improve pregnancy outcomes (18–20). As shown in Figure 2, the proportion of DX5+/CD25+/FOXP3+/CD45+/CD4+-positive cells in the model group was significantly lower than that in the control group (P<0.05); the proportion of positive cells was significantly higher in the VitD3 and progesterone groups than in the model group (P<0.05). Relative to the model group, the DX5+/CD25+/FOXP3+/CD45+/CD4+ proportion in the aspirin group was not significantly increased. The proportion of positive cells in the progesterone group was the most significantly increased from the model group, but no obvious difference was detected between the progesterone group and the control group.

### Detection of Th cells in peripheral blood

**Figure 3:**
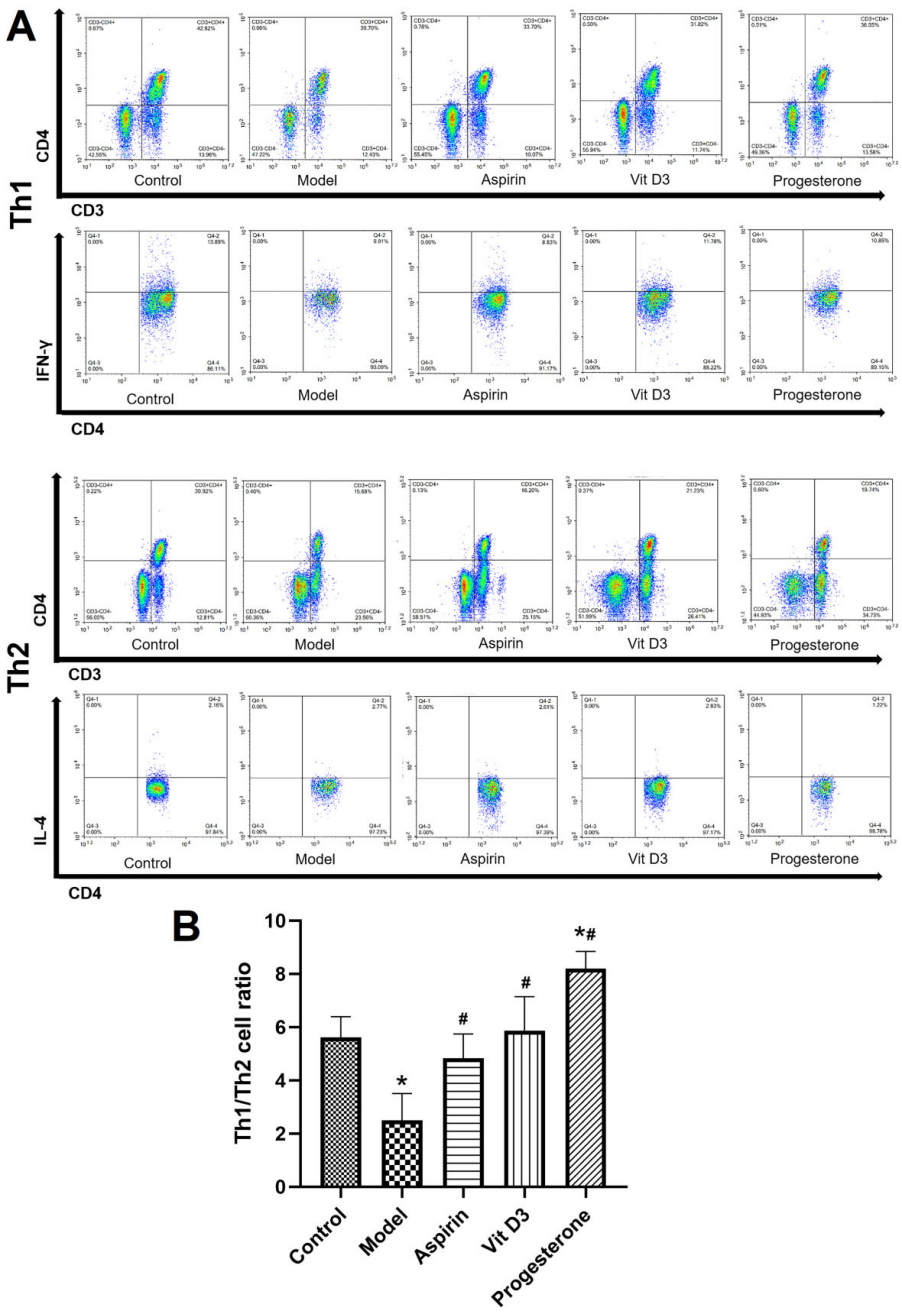
Th1 and Th2 cells in the peripheral blood in a mouse model of recurrent spontaneous abortion treated with aspirin, vitamin D3, and progesterone were analyzed by flow cytometry. **A**, Representative flow cytometry results. **B**, Quantitative analysis of the percentage of Th1 and Th2 cells in each group. Data are reported as means±SD (n=3). *P<0.05 *vs* the control group; ^#^P<0.05 *vs* the model group (ANOVA).

The Th1/Th2 balance is directly related to pregnancy outcomes; therefore, Th1 and Th2 cells in the peripheral blood were analyzed by flow cytometry. As shown in Figure 3, among CD3+/CD4+ positive cells, IFN-γ-positive cells were Th1 cells, and IL-4-positive cells were Th2 cells. The ratio of Th1/Th2 was significantly decreased in the autoimmune RSA model group, indicating an imbalance of Th1/Th2, which was improved by treatment with aspirin, VitD3, and progesterone. The progesterone treatment had the best effect, and the Th1/Th2 ratio was even better than that in the normal control group.

### Detection of serum ACA and &mac_bgr;2-GP1 antibody levels

**Figure 4:**
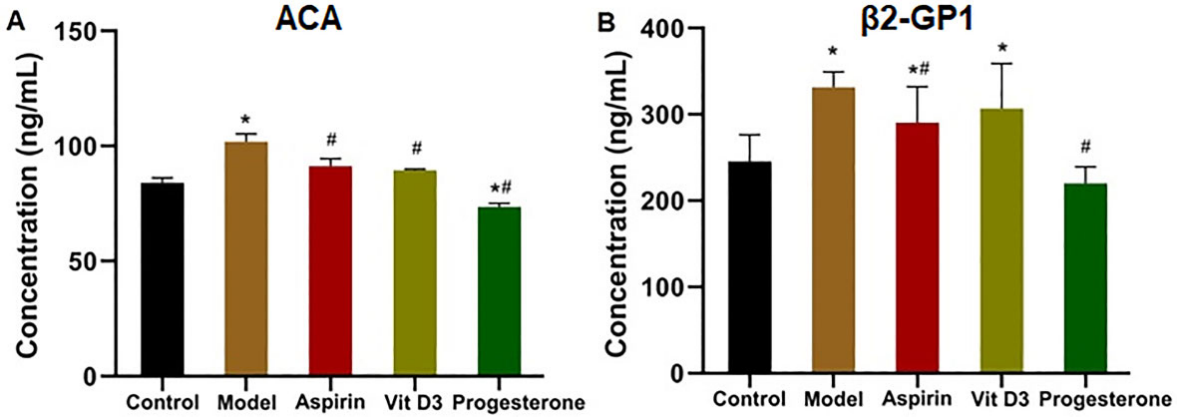
The anti-cardiolipin antibody (ACA) and β2-GP1 levels in a mouse model of recurrent spontaneous abortion treated with aspirin, vitamin D3, and progesterone were detected by ELISA. Data are reported as means±SD (n=3). *P<0.05 *vs* the control group; ^#^P<0.05 *vs* the model group (ANOVA).

High expression levels of ACA and β2-GP1 have been reported to be closely associated with RSA (21). As shown in Figure 4, the ACA and β2-GP1 levels in the model group were significantly higher than those in the control group (P<0.05); the antibody levels in the three treatment groups were lower than those in the model group, and the levels in the progesterone group were significantly lower than those in the model group (P<0.05).

### Detection of serum CXCL6, IFN-&mac_ggr;, and IL-6 cytokines levels

**Figure 5:**
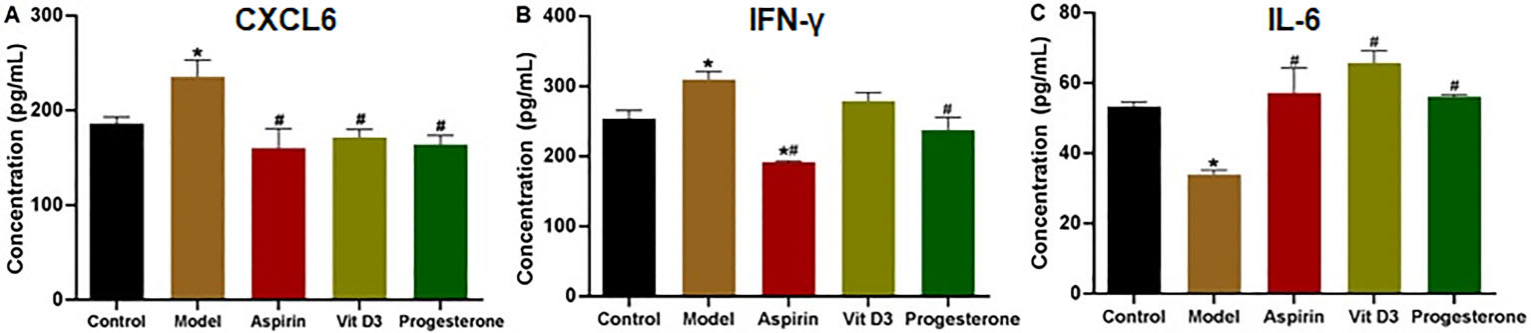
The cytokines in the serum of a mouse model of recurrent spontaneous abortion treated with aspirin, vitamin D3, and progesterone were detected using ELISA. The CXCL6, interferon (IFN)-γ, and interleukin (IL)-6 levels in the serum in each group were analyzed by ELISA. Data are reported as means±SD (n=3). *P<0.05 *vs* the control group. ^#^P<0.05 *vs* the model group.

To evaluate the Th1/Th2 balance, Th1 cytokines (CXCL6 and IFN-γ) and a Th2 cytokine (IL-6) were detected in serum using ELISA (22,23). As shown in Figure 5, the CXCL6 and IFN-γ levels in the model group were significantly lower than those in the control group (P<0.05). Compared with the model group, the CXCL6 and IFN-γ levels in the three medication groups had the most significant decreases in the cytokines measured (P<0.05). The IL-6 protein level in the model group was significantly lower than that in the control group (P<0.05), while the IL-6 levels in the medication groups were significantly higher than those in the model group (P<0.05).

## Discussion

The pathogenesis of autoimmune RSA remains incompletely understood at present, although it is generally believed to be related to the expression of multiple antibodies (24,25). Currently, there are multiple exploratory treatments, but the results of these treatments are partially contradictory. In this study, aspirin, VitD3, and progesterone were used to intervene in the autoimmune RSA pregnancy outcome, and the effects of these three drugs were compared between groups.

First, the DX5+/CD25+/FOXP3+/CD45+/CD4+ expression in placental lymphocytes was detected through flow cytometry. CD45 is a leukocyte common antigen and is generally used as a leukocyte marker (26). CD4+ FOXP3+ T regulatory (Treg) cells are key players in suppressing autoimmunity and maintaining self-immune tolerance (27). DX5+ and CD25+ represent NK cell subsets that contribute to the establishment of immune tolerance (18). A high proportion of these cells is beneficial for maintaining immune tolerance and improving pregnancy outcomes (18–20). In our current study, we found that the VitD3 and progesterone groups had significantly higher proportions of DX5+/CD25+/FOXP3+/CD45+/CD4+ cells than the model group, and progesterone had a better effect than VitD3.

At present, the Th1/Th2 balance is generally believed to be crucial in establishing immune tolerance at the maternal-embryonic interface. Researchers have found a shift in the Th1/Th2 equilibrium towards Th1 dominance in patients with recurrent miscarriage (28). Th1 cells mainly produce proinflammatory cytokines such as tumor necrosis factor-α, IFN-γ, and IL-2, and these cytokines can stimulate cellular inflammation and participate in cellular immunity and the rejection process (29). Conversely, the Th2 cytokines, mainly IL-4, IL-6, IL-10, etc., activate the production of B-cell antibodies and function antagonistically to Th1 cytokines. Th2 cells can act as mediators of humoral immunity and participate in immune tolerance at the maternal-embryonic interface (30,31). Th1 cytokines are dominant in autoimmune RSA (32). The positive expression of DX5+/CD25+/FOXP3+/CD45+/CD4+ in cells was significantly elevated after treatment with progesterone or VitD3, indicating an increase in Th2 cells and that the Th1/Th2 imbalance was improved compared with that of the model group. The increased Th2 cells could enhance immune tolerance, which could ameliorate autoimmune RSA.

Furthermore, the ACA, β2-GP1, CXCL6, IFN-γ, and IL-6 levels in serum were detected by ELISA. ACAs are a group of negatively charged antiphospholipid antibodies; according to prior studies, the ACA positive rate in RSA patients was remarkably higher than that in healthy pregnant women, and ACA can bind with platelet phospholipids, which decreases release of prostacyclin and results in thrombosis (33). Based on our experimental results, three drugs could partially reduce ACA levels and suppress thrombosis. β2-GP1, a single strand glycoprotein, is abundant in plasma and can bind with plasma lipoproteins to expose a concealed epitope, which leads to the production of anti-β2-GP1 antibody. The anti-β2-GP1 antibody has been experimentally verified to suppress the endogenous coagulation pathway and inhibit prothrombinase activity on platelets, giving rise to RSA (34). According to our results, aspirin and progesterone notably suppressed the anti-β2-GP1 antibody. The levels of Th1 factors CXCL6 and IFN-γ in the medication groups were lower than those in the model group, while those of Th2 factor IL-6 were elevated, suggesting that Th2 cells were increased and immune tolerance was enhanced (35,36). Consequently, aspirin, VitD3, and progesterone had various impacts and therapeutic effects on the autoimmune RSA model, and progesterone had the best effect.

At present, the main treatment for autoimmune RSA is heparin combined with aspirin. In addition to anticoagulation, heparin can also suppress the binding of β2-GP1 with the anti-phospholipid antibody, and aspirin can limit the production of thromboxane A2 and relieve vasoconstriction (37,38). In our current study, we found that progesterone alone had the best effect on reducing the embryo loss rate and improving the Th1/Th2 balance. Furthermore, in the autoimmune RSA mice, the plasma ACA, β2-GP1, CXCL6, and IFN-γ levels were significantly higher and the IL-6 level was lower than in the control mice. All these changes were reversed by aspirin and progesterone treatment. Previous study has found that a combination treatment consisting of oral prednisone 20 mg/day and progesterone 20 mg/day for the first 12 weeks of gestation, and aspirin 75 mg/day for 32 weeks of gestation could improve the pregnancy outcomes (39). However, there was not enough evidence to show the effect of aspirin/progesterone or aspirin/vitamin D combination on the pregnancy outcomes. In addition, compared with aspirin, progesterone has widely been used clinically for the treatment of gynecological diseases with little toxicity and few side effects. Further studies will focus on the safety and efficacy of the combination of aspirin and progesterone or VitD3 on autoimmune RSA.
